# Controlling the wavefront aberration of a large-aperture and high-precision holographic diffraction grating

**DOI:** 10.1038/s41377-025-01785-2

**Published:** 2025-03-05

**Authors:** Wenhao Li, Xinyu Wang, Bayanheshig, Zhaowu Liu, Wei Wang, Shan Jiang, Yubo Li, Shuo Li, Wei Zhang, Yanxiu Jiang, Zheng Wu, Wenyuan Zhou

**Affiliations:** 1 Changchun Institute of Optics, Fine Mechanics and Physics, Chinese Academy of Sciences, 130033 Changchun, Jilin China; 2 University of Chinese Academy of Sciences, 100049 Beijing, China

**Keywords:** Lithography, Imaging and sensing

## Abstract

The scanning interference field exposure technique is an effective method to fabricate holographic diffraction grating with meter-level size and nano-level precision. The main problems of fabricating large-aperture and high-precision grating by this technique are the high-precision displacement measurement of the stage, the high-precision control of the interference fringe and the real time compensation of the grating phase error. In this paper, the influence of grating groove error on the wavefront aberration is analyzed. In order to improve the precision of the stage with displacement range more than one meter, an integrated displacement measurement combining grating sensing and laser interferometry is proposed, which suppresses the influence of environment on measurement precision under long displacement range. An interference fringe measurement method is proposed, which combines the diffraction characteristics of the measuring grating with the phase-shifting algorithm. By controlling the direction, period and phase nonlinear errors of the interference fringe, high quality interference fringe can be obtained. Further, a dynamic phase-locking model is established by using heterodyne interferometry to compensate grating phase error caused by stage motion error in real time. A grating with the aperture of 1500 mm × 420 mm is fabricated. The wavefront aberration reaches 0.327*λ* @ 632.8 nm and the wavefront gradient reaches 16.444 nm/cm. This research presents a novel technique for the fabrication of meter-level size and nano-level precision holographic grating, which would further promote the development of chirped pulse amplification systems, high-energy laser and ultra-high precision displacement measurement.

## Abstract (web summary)

Study on wavefront control of holographic grating. This research presents a novel technique for the fabrication of meter-size and nano-precision holographic gratings.

## Graphical abstract

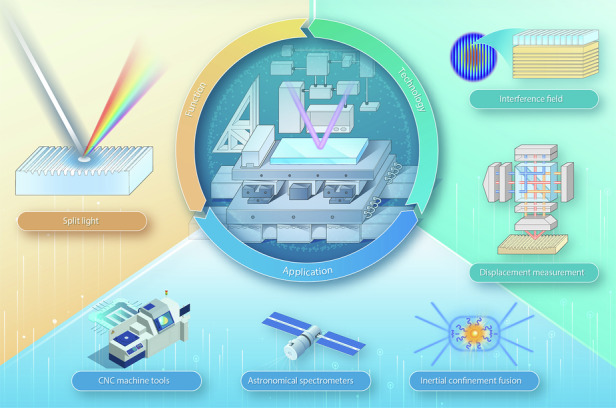

## Introduction

Diffraction gratings, with their unique capability to split light and shift phase, serve as essential beam-modulating components across diverse fields, such as laser modulation^1,2^, spectral analysis^3–5^, displacement sensing^6–8^, biomedicine^9,10^, computational display^11–13^, and ecology^14,15^. They are used as core devices in advanced precision technology such as inertial confinement fusion, astronomical spectrometers, high-end lithographic machines, computer numerical control machine tools (CNC), holographic 3D displays, and biomedical devices^16–22^.

For inertial confinement fusion, large-aperture pulse compression gratings are the key to realize the ultra-short pulse and ultra-high-power laser in chirped-pulse amplification systems. The wavefront aberration induced by grating groove error can induce wavefront deformation after beam combination, resulting in spot diffusion and laser performance degradation^23–25^. For astronomical spectrometers, the weak receiving signal intensity from detected targets requires a large-aperture and high-precision holographic grating to improve the light throughput and signal-to-noise ratio^26–30^. Consequently, the grating groove error would exacerbate the wave aberration in the exit pupil, ultimately reducing spectral resolution and limiting system detection. In precision displacement measurement, grating sensing systems serve as core components in CNC machine tools, directly impacting processing accuracy. The grating groove error distorts the wavefront, resulting in a decrease in displacement measurement precision^31–34^. Therefore, in the grating fabricating process, precise control of the grating groove error is crucial to achieve high-quality diffraction wavefront^35–38^.

The Lawrence Livermore National Laboratory in the United States has fabricated a 910 mm × 455 mm aperture grating with a +1^st^-order wavefront aberration of 0.255*λ* @1053 nm^39,40^ by static exposure system. It requires large aperture optical elements for beam shaping, and the cost of fabricating large holographic grating is enormous. The Plymouth Grating Laboratory has fabricated a grating with the aperture of 920 mm × 420 mm and wavefront aberration of *λ*/3 @ 632.8 nm^41,42^, it is used for the long-pulse compressors in the LFEX laser system. The University of Science and Technology of China has fabricated a large-aperture grating with the aperture of 1400 mm × 420 mm and wavefront aberration of less than 0.5*λ* @ 632.8 nm by splicing three sub-gratings^43^. Soochow University has fabricated the grating with the aperture of 80 mm × 110 mm by double-exposure splicing and achieved an alignment error between adjacent exposure areas less than 0.5*λ* @ 632.8 nm^44^. Tsinghua University has fabricated a 1 × 2 spliced grating using multiple-exposure splicing, achieving a −1^st^-order wavefront aberration of better than 0.1*λ* @ 632.8 nm within a 90 mm × 160 mm aperture^45^. However, seams are obvious for the spliced grating, so the wavefront aberration of the full aperture grating is very poor. In summary, there is currently no report work on high-precision and seamless grating with the aperture greater than one meter, which hinders the advancements in spectroscopy, laser systems, and related fields. Therefore, in order to achieve low wavefront aberration of meter-level size and nano-level precision holographic grating, it is urgent to develop novel methods of controlling the grating error.

The main causes of wavefront aberration consist of the substrate surface error and grating groove error. The high-quality substrate surface error is reliant on processing capability, so achieving high-precision holographic grating requires tight control over groove error. However, in a static exposure system used to fabricate the holographic grating, groove error is determined by the collimator and cannot be active controlled in real time^46^. Therefore, the scanning interference field exposure system will open up a method for fabricating high-precision and large-aperture grating^47^. In this technique, two small Gaussian laser beams are superimposed at the waist of the beam to form interference fringe. The two-dimensional stage carries out step-scan motion, and the interference fringe is spliced onto the grating substrate by the scanned mode^48,49^. Therefore, the main challenges to achieve low wavefront aberration of meter-level size holographic grating of this method are the high-precision displacement measurement of the stage, the high-precision control of the interference fringe and the real time compensation of the grating phase error.

In this work, we have fabricated a complete set of the scanning interference field exposure system. This system addresses key challenges, including the high-precision displacement measurement of the stage, the high-precision control of the interference fringe and the real time compensation of the grating phase error. A grating with the aperture of 1500 mm × 420 mm and the wavefront aberration of 0.327*λ* @ 632.8 nm is achieved by this system. This achievement overcomes the longstanding challenge of simultaneously attaining large aperture and high precision in grating production. The results verify this method as contributes an alternative for fabricating large-aperture and high-precision grating.

## Results

**Fig. 1:**
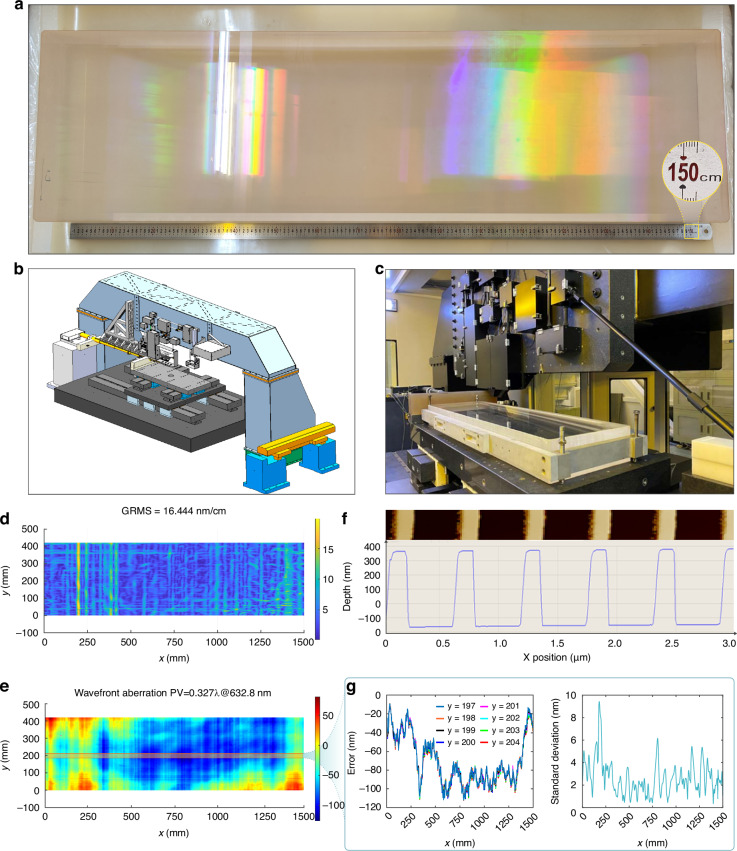
Results of grating with the aperture of 1500 mm × 420 mm fabricated by the scanning interference field exposure system. **a** Photograph of the holographic grating with the aperture of 1500 mm × 420 mm. **b** Schematic of the scanning interference field exposure system. **c** Photograph of the scanning interference field exposure system. Results of the fabricated grating with **d** wavefront gradients root mean square and **e** +1^st^-order wavefront aberration. **f** Grating groove profile measured by AFM. **g** Error of grating groove error and standard deviation of groove error

As shown in Fig. 1a–c, we have fabricated the scanning interference field exposure system with the exposure region of 1700 mm × 650 mm, which is successfully used to fabricate the holographic grating with a groove period of 1740 groove per millimeter (gr/mm) and the aperture of 1500 mm × 420 mm. Figure 1d exhibits the fabricated grating with the wavefront gradient is 16.444 nm/cm. Moreover, +1st-order wavefront aberration of 0.327λ @ 632.8 nm under Littrow incidence and the groove error at different positions of grating presents good consistency, as shown in Fig. 1e, g. Grating groove profile measured by AFM as shown in Fig. 1f.

## Discussion

**Fig. 2:**
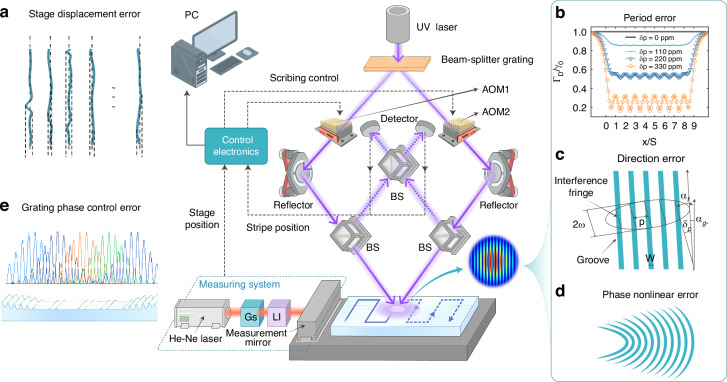
Influence of different errors in the scanning interference field exposure system. The solid line represents the propagation path of the exposure light beam, while the dotted line represents the propagation path of the light beam in the interference fringe measurement system. **a** Grating groove error caused by stage displacement error. Interference fringe error including **b** Period error, **c** Direction error and **d** Phase nonlinear error. **e** Grating phase control error

The schematic diagram of the scanning interference field exposure system independently built by CIOMP is shown in Fig. 2. The principle is that two Gaussian laser beams from Ultraviolet laser (UV laser) are superimposed at the waist of the beam through the optical system to form interference fringe, and then the interference fringe is recorded on the substrate coated with photoresist by using a two-dimensional displacement stage in the way of step-by-step scanning. Multiple errors of the scanning interference field exposure system during the exposure would influence the precision of grating, including stage displacement error (Fig. 2a), interference fringe error (Fig. 2b–d), and grating phase control error (Fig. 2e).

In order to achieve the high-precision control of wavefront aberration, the grating wavefront modulation model is established, and the effect of grating groove error on grating wavefront is analyzed.

In order to measure the position of grating substrate accurately, an integrated displacement measurement combining grating sensing and laser interferometry is proposed. The repeatability measurement precision of the stage reaches ±6 nm.

To obtain high-quality interference fringe, the direction, period and phase nonlinear errors of the interference fringe is measured and controlled to reduce the influence of the interference fringe morphology on exposure contrast and grating groove error. The direction error of the interference fringe is adjusted to 0.677 μrad by beam alignment. The repeatability of period measurement reaches 0.42616 ppm by dual-frequency laser interferometer. The PV value of phase nonlinear error within the effective light spot is 21.8 nm.

To achieve the required precision of grating groove error, a dynamic phase-locking model based on heterodyne interferometry theory is established and optical modulator method is used to implement the measurement and feedback of groove error to real time compensate grating phase error caused by stage displacement error. The 3*σ* value of the fringe phase after static and dynamic locking is 0.349 rad and 0.355 rad, respectively.

## Materials and methods

### Model for controlling the grating groove error

The scanning interference field exposure system uses two Gaussian beams to form equal periodic interference fringe on a grating substrate placed on a two-dimensional displacement stage. The stage moves in steps mode along the long side of the grating substrate (defined as the X direction) and in scan mode along the short side of the grating substrate (defined as the Y direction). Grating grooves would be formed by step-by-step splicing interference fringe with the movement of stage in the X direction. Therefore, stage displacement error and interference fringe error would directly determine the grating groove error, and then affect the wavefront aberration of the grating. In this part, the relationship among the grating groove error, stage displacement error and interference fringe error is analyzed, which lays a theoretical foundation for controlling the wavefront aberration of a large-aperture and high-precision holographic grating.

The grating wavefront aberration ${W}_{rn}$ which exists substrate surface error and grating groove error can be expressed as^50^:

1 $${W}_{rn}=S(\cos {\theta }_{1}+\,\cos {\theta }_{2})+G\frac{m\lambda }{p}$$

where $S$ is substrate surface error, ${\theta }_{1}$ and ${\theta }_{2}$ are incidence angle and the diffraction angle, respectively, *G* is the grating groove error, $m$ is the diffraction order of grating, $\lambda$ is the wavelength of incident laser, $p$ is the grating period.

The incident laser is split into two beams and each beam is treated as plane wave with a Gaussian profile. Two split beams propagate in a symmetrical optical path and interfere with each other at the waist of the beam. The interference intensity distribution $I(x,y,t)$ is expressed as:

2 $$I(x,y,t)=\frac{{A}_{1}^{2}+{A}_{2}^{2}}{2}\exp \left(-2\frac{{x}^{2}+{y}^{2}}{{w}^{2}}\right)\left[1+\frac{2{A}_{1}{A}_{2}}{{A}_{1}^{2}+{A}_{2}^{2}}\,\cos \varphi (x,t)\right]$$

where ${A}_{1}$ and ${A}_{2}$ are the amplitude of the two beams, respectively, $w$ is the spot radius of the two beams at the waist of the beam, $\varphi (x,t)$ is the phase of interference fringe.

The angle between the exposure beam and the grating normal is $\theta$ and optical path of left and right beam from the beam-splitter grating to the exposure area are ${L}_{1}$ and ${L}_{2}$, respectively. Therefore $\varphi (x,t)$ is expressed as:

3 $$\varphi (x,t)=2x\,\sin \theta ({k}_{1}+{k}_{2})+({k}_{2}{L}_{2}-{k}_{1}{L}_{1})+2\pi ({f}_{1}-{f}_{2})t+{\varphi }_{n}(x)$$

where ${k}_{1}=2\pi /{\lambda }_{1}$ and ${k}_{2}=2\pi /{\lambda }_{2}$ are wave numbers with two beams wavelengths ${\lambda }_{1}$ and ${\lambda }_{2}$, ${f}_{1}$ and ${f}_{2}$ are the frequencies of left and right beams, and *φ*_*n*_ is phase nonlinear error of the interference fringe.

Define the scanning path of the *n*^th^ scan in the *y* direction parallel to the interference fringe be $x={S}_{n}(y)$, then the exposure amount $D(x,y)$ of the photoresist surface on the grating substrate is the sum of the exposures of $N$ scans:

4 $$D(x,y)=\mathop{\sum }\limits_{n=1}^{N}{\int }_{-\infty }^{+\infty }I(x,y,t)dt\propto \,\cos ({\varPhi }_{e}(x,y))$$

${\varPhi }_{e}$ is the exposure phase distribution after *N* scans. It is determined by the groove error of the *n*^th^ scan and the weighting proportion of the adjacent groove error. Assuming the step distance is 0.9*w*, the proportion of the adjacent groove error is negligible, ${\varPhi }_{e}$ is approximated as the exposure phase distribution of the *n*^th^ scan:

5 $${\varPhi }_{e}(x,y)=2\pi \left(\frac{x}{p}+\left(\frac{{L}_{2}}{{\lambda }_{2}}-\frac{{L}_{1}}{{\lambda }_{1}}\right)+{\varphi }_{ph}+{\varphi }_{s}\right)$$

where $p=\lambda /2\,\sin \theta$ is the grating period, $({L}_{2}/{\lambda }_{2}-{L}_{1}/{\lambda }_{1})$ is determined by the optical path difference of the two beams, ${\varphi }_{ph}$ is the phase term related to the frequency modulation of the beam and time, which is what the phase control system adjusts, ${\varphi }_{s}$ is the phase change due to phase nonlinear error. Further, ${\varphi }_{ph}$ and ${\varphi }_{s}$ could be expressed as:

6 $$\left\{\begin{array}{c}{\varphi }_{ph}=\left(({f}_{1}-{f}_{2})t-\frac{{S}_{n}(y)}{p}\right)\\ {\varphi }_{s}=\arctan \displaystyle\frac{\mathop{\int }\nolimits_{-\infty }^{+\infty }\exp \left(-\displaystyle\frac{2{y}^{2}}{{w}^{2}}\right)\sin {\varphi }_{n}(x,\,y)dy}{\mathop{\int }\nolimits_{-\infty }^{+\infty }\exp \left(-\displaystyle\frac{2{y}^{2}}{{w}^{2}}\right)\cos {\varphi }_{n}(x,\,y)dy}\end{array}\right.$$

According to ${\varphi }_{ph}$ in the Eq. (5), the position of the grating groove could be adjusted by controlling the frequency difference between the two beams, so as to compensate the substrate position error and the optical path difference between the beams. Acousto-optic modulator (AOM) is therefore inserted into the optical paths on both sides to adjust the beam frequency. When the modulation frequencies of the two AOMs (AOM1 and AOM2 in Fig. 2) are set to${f}_{1v}$ and ${f}_{2v}$ respectively, the frequencies of left and right beams correspond to ${f}_{1}={f}_{0}+{f}_{1v}$ and ${f}_{2}={f}_{0}+{f}_{2v}$ respectively. Therefore, the frequency difference between the two coherent beams is ${f}_{1v}-{f}_{2v}$, which could be controlled by AOMs to adjust the position of the grating groove. If there is an angle *α* between the direction of the interference fringe and the direction of scanning motion is *α*, ${\varphi }_{ph}$ would be expressed as:

7 $${\varphi }_{ph}=({f}_{1v}-{f}_{2v})t-\frac{{S}_{n}(y)}{p(1+\Delta p)}\cos \alpha$$

According to Eq. (7), the production of high-precision grating requires high-precision displacement measurement of stage and high-precision control of interference fringe.

### Integrated displacement measurement system combining grating sensing and laser interferometry

At present, dual-frequency laser interferometer is the most commonly setup for the displacement measurement of two-dimensional stage. However, due to the measurement basis of laser wavelength, the wavelength changes with the fluctuations of non-vacuum environmental factors such as temperature and pressure, which affects the measurement accuracy and leads to the decline of the exposure quality. Under the same environmental conditions, the grating sensing could achieve higher measurement stability than the laser interferometer because of its shorter optical path. However, the grating sensor is not suitable for measuring the displacement of a two-dimensional step-scanning stage because the position feedback of the tested object is received by a probe that rigidly connected to the stage in the stepping direction which cannot measure the displacement in the scanning direction. To solve these problems, integrated displacement measurement system combining grating sensing and laser interferometry is proposed. Grating sensing with long range measurement is carried out far from the mirror, and laser interferometry with short range measurement is carried out near the mirror, which thereby suppresses the influence of environment on measurement precision under long displacement range.

**Fig. 3:**
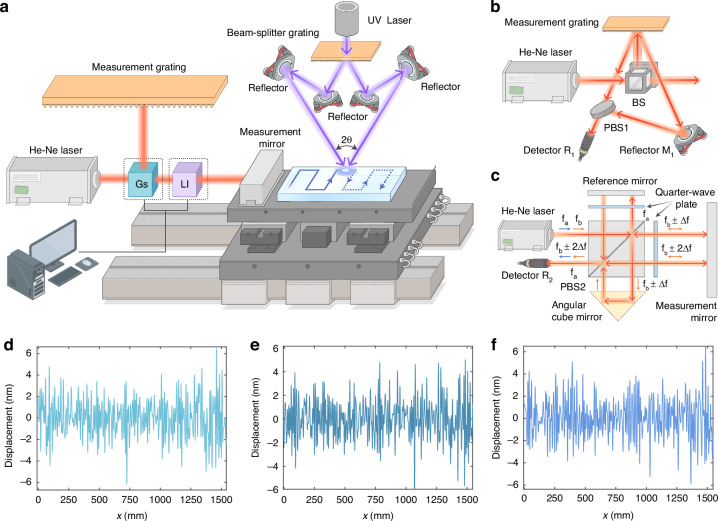
**a** Schematic of the integrated displacement measurement system combining grating sensing and laser interferometry. **b** Schematic of the grating sensing. **c** Schematic of the laser interferometer. Step displacement errors at different scanning positions **d** 0 mm, **e** 150 mm and **f** 300 mm along Y direction

Figure 3a shows the principle of integrated displacement measurement system combing grating sensing and laser interferometry. The beam splitter (BS) is used to split the beam of orthogonal and linearly polarized light with certain frequency difference emitted by the dual-frequency laser. The reflected light is diffracted by the measurement grating. The +1^st^-order diffracted light with frequency ${f}_{a}$ is reflected by ${M}_{1}$ onto a polarizing beam-splitter prism (PBS), and then reflected by PBS1, enter the detector ${R}_{1}$. The −1^st^-order diffracted light with frequency ${f}_{b}$ is incident into PBS1, and then transmitted through PBS1. The beam reflected by PBS1 would interfere with the beam transmitted through PBS1, as shown in Fig. 3b. According to the Doppler principle, when the probe moves, the phase change $\Delta {\phi }_{g}$ in the interference signal caused by grating sensing is:

8 $$\Delta {\phi }_{g}=2\pi \Delta {f}_{g}=\frac{4\pi }{d}{\int }_{0}^{t}v(t)dt=\frac{4\pi }{d}\Delta {x}_{g}$$

where $\Delta {f}_{g}$ is the Doppler frequency shift caused by the movement of the probe, $v(t)$ is the moving speed of the stage as a function of time, and $\Delta {x}_{g}$ is the displacement of grating sensing. $\Delta {x}_{g}$ could be expressed as:

9 $$\Delta {x}_{g}=\frac{d}{4\pi }\Delta {\phi }_{g}=\frac{d}{2M}{C}_{g}$$

where *d* is grating period, ${C}_{g}$ is the displacement value output by grating subdivision system, and *M* is the number of electronic subdivisions.

After the transmitted light from He-Ne laser passes through the PBS2, the beam with frequency ${f}_{b}$ propagates through a quarter-wave plate and reaches the measurement mirror. The reflected beam passes through the quarter-wave plate again to return to PBS2 and then to the angular cube mirror. The beam passes through PBS2 and the quarter-wave plate again to reach the measurement mirror. The beam reflected by the measurement mirror is returned to quarter-wave plate and PBS2, and then received by the detector ${R}_{2}$. Similarly, the transmitted beam from He-Ne laser with frequency ${f}_{a}$ is received by the same detector, as shown in Fig. 3c. The interference signal would be formed by two beams of frequency ${f}_{a}$ and ${f}_{b}$ interfering with each other. According to the Doppler principle, the phase change $\Delta {\varphi }_{i}$ of the interference signal caused by the movement of the measuring mirror is:

10 $$\Delta {\phi }_{i}=2\pi \Delta {f}_{i}=\frac{2\pi f}{c}{\int }_{0}^{t}v(t)dt=\frac{2\pi }{{\lambda }_{l}}\Delta {x}_{i}$$

where $\Delta {f}_{i}$ is the Doppler frequency shift, $c$ is the light speed, $f$ is the central frequency of the He-Ne laser, ${\lambda }_{l}$ is the central wavelength of the He-Ne laser, and $\Delta {x}_{i}$ is the displacement of the laser interferometer. $\Delta {x}_{i}$ could be expressed as:

11 $$\Delta {x}_{i}=\frac{{\lambda }_{l}}{2\pi }\Delta {\phi }_{i}=\frac{{\lambda }_{z}}{4{n}_{0}M}{C}_{l}$$

where ${C}_{l}$ is displacement value output by laser subdivision system, ${\lambda }_{z}$ is the He-Ne laser wavelength in vacuum, and ${n}_{0}$ is the refractive index of air.

The displacement $\Delta x$ of stage could be expressed as:

12 $$\Delta x=\Delta {x}_{g}+\Delta {x}_{i}=\frac{d}{4\pi }\Delta {\phi }_{g}+\frac{\lambda }{2\pi }\Delta {\phi }_{i}=\frac{d}{2M}{C}_{g}+\frac{{\lambda }_{z}}{4{n}_{0}M}{C}_{l}$$

The grating sensing and the laser interferometer use the same set of counting and acquisition boards, ensuring the synchronous collection of data. The grating with the aperture of 1500 mm × 420 mm requires more than 10 h of continuous exposure. We adopted to simultaneously record the linear displacement of the stage at absolute positions (0, 150, and 300 mm) every 1 h, and a total of 10 groups of measurement data were recorded to verify the measurement accuracy of the stage moving 1500 mm in the X direction. As shown in Fig. 3d–f, we used short-time laser interferometer data as a baseline, the difference between the proposed integrated displacement measurement system combining grating sensing and laser interferometer was kept within ± 6 nm.

### Measurement and adjustment for the period and direction of interference fringe

**Fig. 4:**
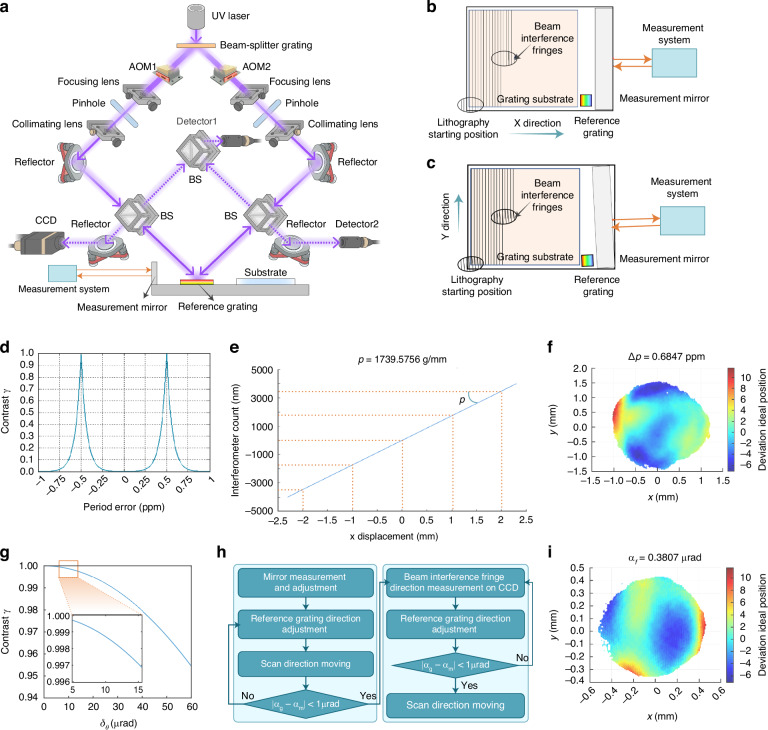
Principle for measuring interference fringe period and direction. The solid line represents the propagation path of the exposure light beam, while the dotted line represents the propagation path of the light beam in the interference fringe period and direction measurement system. **a** The experimental optical path diagram of interference fringe period and direction. **b** Schematic diagram of interference fringe period error. **c** Schematic diagram of interference fringe direction error. **d** Relation between the exposure contrast and period error. **e** Reference grating groove period single measurement data fitting. **f** Interference fringe period and reference grating period error$\Delta p$. **g** Relation between the exposure contrast and the angle between the interference fringe direction and scanning direction. **h** Flow chart of interference fringe direction adjustment procedure. **i** Angle between the interference fringe direction and the groove direction of the reference grating

The interference fringe formed by two Gaussian beams from UV laser is spliced to fabricate the large aperture grating by step scanning motion. Interference fringe period error (Fig. 4b) and direction error (Fig. 4c) are important factors affecting grating quality. Figure 4d simulates the relationship between interference fringe period error and interference fringe contrast. When the period error reaches the order of 2 ppm, the effects on the shape and error of the grating groove are both negligible.

The measurement method of interference fringe period error is proposed to obtain the error of interference fringe period error less than 2 ppm by combining the difference frequency measurement and phase-shifting algorithm of the reference grating. This method is realized by measuring and adjusting the period error between the reference grating and the interference fringe. The measurement principle is shown in Fig. 4a. The reference grating with the same groove density as the fabricated grating is placed on the plane of the grating substrate. The diffraction of the two exposure beams through the reference grating satisfies the Littrow condition, that is, the incident beam is coincide with the +1^st^ or -1^st^-order diffracted beam. At the same time, part of the beam separated from the two optical paths passes through the BS and then is overlapped into detector 1 as a phase reference signal. The left beam through the reference grating generates -1^st^-order diffraction beam. and the reflected beam generated by the right beam through the BS prism sampling into detector 2, as a phase measurement signal. So, detector 1 and detector 2 will receive the reference phase of interference fringe and the measurement phase of reference grating information, respectively. By moving the reference grating in the X direction, the phase information changes of the two detectors are compared with the phase changes of the dual-frequency laser interferometer. The reference grating period ${p}_{m}$ is obtained:

13 $${p}_{m}=\frac{2\pi }{\Delta {\varphi }_{{\rm{L}}}}\Delta x$$

where $\Delta {\varphi }_{L}$ is the phase change caused by the displacement change in X direction of the reference grating, $\Delta x$ is the displacement change of laser interferometer. The interference fringe of the exposure beam on the substrate surface can be written as:

14 $$\begin{array}{ll}I={I}_{a}(x,y)\cos \left\{\frac{2\pi }{{p}_{m}}\left[1+\frac{\cos \theta }{2\,\sin \theta }\cdot ({\delta }_{Lx}-{\delta }_{Rx})\right]x\right.\\\quad\;\;\,+\,\left.\frac{2\pi }{\lambda }({\delta }_{Ly}-{\delta }_{Ry})y+{\varphi }_{0}\right\} +{I}_{b}(x,y)\end{array}$$

where, $\theta$ represents the incident angle of the exposure beam, ${\delta }_{Lx}$,${\delta }_{Ly}$ denote the deviation angle of the left beam from the incident direction, ${\delta }_{Rx}$,${\delta }_{Ry}$ represent the deviation angle of the right beam from the incident direction, ${I}_{{\rm{a}}}(x,y)$ and ${I}_{{\rm{b}}}(x,y)$ are quantities related to the light intensity distributions of the exposure beams on both sides, and ${\varphi }_{0}$ is the phase of the interference fringe at the origin. It can be seen from the above formula that the difference between the period error of the interference fringe and the period error of the reference grating is related to ${\delta }_{Lx}-{\delta }_{Rx}$. Therefore, by adjusting the deviations of the exposure beams on both sides from the incident direction and measuring the period error of the reference grating, the period error of the interference fringe can be obtained. The period error of the interference fringe can be expressed as:

15 $$p=\frac{{p}_{m}}{1+\frac{{\delta }_{Lx}-{\delta }_{Rx}}{2\,\tan \theta }}$$

Charge-coupled device (CCD) is placed symmetrically on the left optical path, so that +1^st^ -order diffraction light and the right reflected light from the left beam remerge into the surface of the CCD. The two beams have the same frequency and the -1^st^ -order diffraction light carrying the phase information of the reference grating can be analyzed by using the interference pattern received by the CCD. Through the Hariharan five-step phase-shifting algorithm, by analyzing the phase distribution of the interference fringe on the CCD surface, the ${\delta }_{Lx}-{\delta }_{Rx}$ can be obtained. Substituting it into formula (15), the period error of the interference fringe can be found. In the experiment, the reference grating with groove period 1740 gr/mm was used. As shown in Fig. 4e, the average period of reference grating is 1739.5756 gr/mm. The period error between the interference fringe period and the reference grating period is measured in a single time, as shown in Fig. 4f. In order to avoid the chance of the experimental results, ten sets of period measurements were repeated under the same experimental conditions and the period error is shown in Table 1. The PV value of the period error is 1.3749 ppm and the root mean square of the period error is 0.42616 ppm. In this situation, the effect of the interference fringe period error on the grating groove is negligible.

**Table 1:** Ten sets of the measured period error

Period error (ppm)	**Data1**	**Data2**	**Data3**	**Data4**	**Data5**
	0.68472	−0.69018	0.36626	0.18848	0.01822
	**Data6**	**Data7**	**Data8**	**Data9**	**Data10**
	−0.34612	−0.5912	0.02928	−0.22864	−0.25431

Root mean square $\Delta p$ = 0.42616 ppm

In practice, the interference fringe is not parallel to the scanning direction. If the angle between the fringe direction and the scanning direction can be accurately measured, the relative displacement between the fringe and the substrate during the scanning can be compensated by the dynamic phase locking, and optimal exposure contrast can also be obtained. Using the phase-shifting algorithm of grating diffraction, we propose a method to adjust the fringe direction by using the reference grating. The groove direction of the reference grating is adjusted to be parallel to the scanning direction, so that the reflected light beam passing through the reference grating on one side coincides with the +1^st^-order diffracted light beam passing through the reference grating on the other side. At the same time, this method also considers the measurement and adjustment of the interference fringe period, simplifies the number of optical elements in the scanning interference field exposure system.

The relationship between the exposure contrast and the angle between the interference fringe direction and the scanning direction is analyzed. The exposure contrast gradually decreases as the angle increases for a waist radius $w=0.9$ mm and an interference fringe period $p=556.56$ nm, as shown in Fig. 4g. The fringe contrast gradually decreases as the scanning angle increases. When the angle is less than 10 μrad, the fringe contrast with ±5 μrad yaw are both higher than 0.99. This means that the influence of the fringe direction on the fringe contrast is less than 1%, and the change in fringe contrast caused by $10\pm 5$ μrad yaw is less than 0.005.

The measurement and adjustment process is illustrated in Fig. 4h. First, the angle *α*_*m*_ between the measuring mirror and the scanning direction is adjusted to be close to parallel with the scanning direction. After ensuring the stability of the measuring mirror, the angle *α*_*g*_ between the reference s groove direction and the scanning direction is adjusted, ensuring that the difference between this angle ${\alpha }_{g}$ and the angle ${\alpha }_{m}$ is less than 1 μrad. Next, the interference pattern collected by the CCD is used to determine the angle between the reference grating groove direction and the interference fringe direction ${\alpha }_{f}$. Finally, the angle between the interference fringe direction and the measuring mirror direction is obtained.

The stage moves 50 mm in the Y direction, and synchronously collects the displacement data measured by the X-axis measuring mirror and the phase change of the reference grating. Ten sets of the measured data are shown in Table 2. By linear fitting of the data, the angle between the measuring mirror and the scanning direction is calculated ${\alpha }_{m}=174.3235$ μrad, the angle between the reference grating groove direction and the scanning direction ${\alpha }_{g}=173.4576$ μrad. The phase distribution is obtained by using the phase-shifting algorithm after collecting five phases by CCD, as shown in Fig. 4i. The angle between the direction of the interference fringe and the X-axis measuring mirror is ${\alpha }_{g}+{\alpha }_{f}-{\alpha }_{m}=-0.4852$ μrad.

**Table 2:** Ten sets of the measured direction error

	Data1	Data2	Data3	Data4	Data5
${\alpha }_{m}$ (μrad)	174.2617	174.5062	174.4402	174.3039	174.5676
${\alpha }_{g}$ (μrad)	173.9647	173.5190	173.4635	173.4164	173.4600
	**Data6**	**Data7**	**Data8**	**Data9**	**Data10**
${\alpha }_{m}$ (μrad)	174.3257	174.2123	174.4220	174.3827	173.8128
${\alpha }_{g}$ (μrad)	173.4073	173.4431	173.4626	173.4779	172.9611

Average ${\alpha }_{m}$ (μrad)= 174.3235, Average ${\alpha }_{g}$ (μrad)=173.4576

### Measurement and adjustment for the phase nonlinear error of interference fringe

In the scanning interference field exposure system, the ideal fringe is equally spaced and straight. However, the phase nonlinear error will lead the bend of the interference fringe in practice. The fringe is dynamically scanned and spliced multiple times to form the total exposure. The exposure at each point on the grating is not determined by a single point in the interference field, but is the sum of the exposures of all the light passing through that point. The phase nonlinear error of the interference fringe indirectly affects the exposure contrast and groove error. Therefore, the influence of the phase nonlinear error of the interference fringe on the exposure is analyzed.

**Fig. 5:**
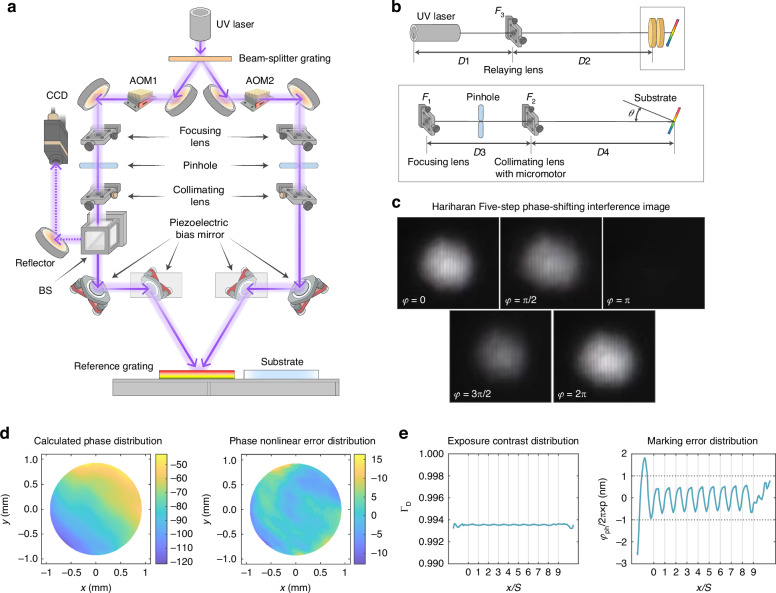
Measurement and adjustment of phase nonlinear error. The solid line represents the propagation path of the exposure light beam, while the dotted line represents the propagation path of the light beam in the phase nonlinear error measurement system. **a** Schematic of the setup for measuring phase nonlinear error. **b** Optical path for phase nonlinear error measurement. **c** Hariharan five-step phase-shifting interference image. **d** Calculated phase distribution of interference fringe after adjustment. **e** Influence of phase nonlinear error of interference fringe on exposure

The schematic of the system for measuring phase nonlinear error is shown in Fig. 5a. The left beam is diffracted from the reference grating. The right beam is reflected by the reference grating. The +1^st^-order diffracted light of the left beam and the 0^th^-order reflected light of the right beam overlap and propagate to the CCD for reception after passing through several plane mirrors. The reference grating only affects the phase constant and propagation direction of the left beam. The propagation paths of the left +1^st^-order diffracted beam and the right reflected beam are the same after the reference grating. The two beams overlap on the CCD, and the interference pattern is formed. The fringe period of the interference pattern detected by the CCD is relatively large, and only bright or dark fringe exist within the entire interference light spot. The phase distribution of the interference pattern can be regarded as the phase nonlinear error in the interference fringe after the slope is removed. This is equivalent to taking one side beam as the reference beam and the other beam as the measurement beam. When the measured phase nonlinear error is used as feedback, the stage is controlled to change the distance between the collimating lens *F*_1_ and the focusing lens *F*_2_, as shown in Fig. 5b, to reduce the phase nonlinear error in the interference fringe.

The collimating lenses of the optical paths on both sides are fixed on the stage and can be axially adjusted as needed. The CCD is used to record the intensities of the interference pattern. The wavefront is restored using the Hariharan five-step phase-shifting algorithm to obtain the distribution of the phase nonlinear error of the interference fringe as shown in Fig. 5c. This error is reduced by adjusting the position of the collimating lens in the optical path. Because the parts of light spot with weak light intensity have little influence on the exposure contrast and groove position error, the effective light spot part with light intensity greater than 1/e^2^ of the central light intensity is selected. The PV value of the selected light spot is 21.8 nm, as shown in Fig. 5d. After the phase nonlinear error distribution is averaged twice through scanning and splicing, the influence on the groove position error is greatly reduced. To verify that the method meets the requirement, the obtained phase nonlinear error in the experiment was used to calculate the exposure contrast and groove position error for a 10-scan exposure were obtained for a step interval of 0.6 mm. The phase nonlinear error of the interference fringe causes that the fringe contrast is decreased from 1 to 0.9935, and the variation of the groove error is approximately 1 nm. The distributions of the groove position error and the fringe contrast are still periodic with the step interval as the period, but the variations are small, as shown in Fig. 5e.

### Measurement and control feedback for high-precision grating phase error

**Fig. 6:**
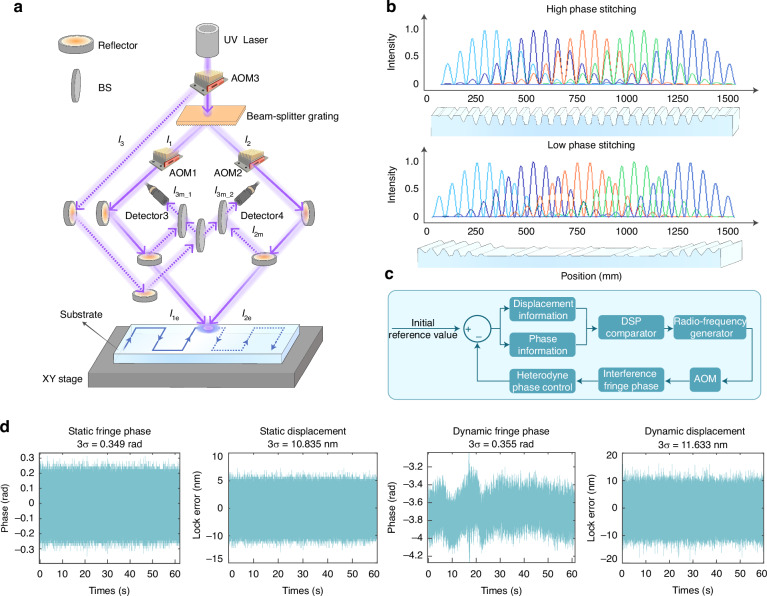
Measurement and control feedback for high-precision grating phase error. The solid line represents the propagation path of the exposure light beam, while the dotted line represents the propagation path of the light beam in the interference fringe position measurement system. **a** Schematic of the system for measuring interference fringe position. **b** Grating fabrication with a splicing error in the phase. **c** Flow chart for fringe control feedback. **d** Phase control feedback data

In order to splice the two exposure phases, it is necessary to obtain the relative positions of the groove after the previous scan and to be exposed in the subsequent scan. If there is a low phase stitching in the phase, the fabrication of grating would fail, as shown in Fig. 6b. Heterodyne interferometry is adopted to ensure that the phase is measured with the same precision as that of the integrated displacement measurement system combining grating sensing and laser interferometry. The measurement principle is shown in Fig. 6a. The fixed-frequency acousto-optic modulator AOM3 is introduced before the beam-splitter grating. After the beam passes through AOM3, the +1^st^-order diffracted light ${I}_{3}$ is divided into two equivalent parts, ${I}_{3m\_1}$ and ${I}_{3m\_2}$, through a BS, and enter the optical path for the position measurement of interference fringe as reference lights. The beams *I*_1_ and *I*_2_ are each divided into two parts through a BS. ${I}_{1e}$ and ${I}_{2e}$ are transmitted onto the upper surface of the grating substrate, and beams ${I}_{1m}$ and ${I}_{2m}$ pass through the optical path for the measurement of fringe position and are received by detector 3 and 4 through a beam-combining device. The light intensities ${I}_{1}$ and ${I}_{2}$ are:

16 $$\begin{array}{c}{I}_{1}={A}_{1m}{A}_{3m\_1}\,\cos 2\pi [({f}_{1}-{f}_{3})t+(\frac{{L}_{3}}{{\lambda }_{3}}-\frac{{L}_{1}}{{\lambda }_{1}})]\\ {I}_{2}={A}_{2m}{A}_{3m\_2}\,\cos 2\pi [({f}_{2}-{f}_{3})t+(\frac{{L}_{3}}{{\lambda }_{3}}-\frac{{L}_{2}}{{\lambda }_{2}})]\end{array}$$

where${A}_{1m}$, ${A}_{2m}$, ${A}_{3m\_1}$, and ${A}_{3m\_2}$ are the intensities of beams ${I}_{1}$, ${I}_{2}$, ${I}_{3m\_1}$, and ${I}_{3m\_2}$ respectively. ${L}_{1}$ and ${L}_{2}$ are the optical paths of the left and right beams from the beam-splitter grating to the exposure area respectively. ${L}_{3}$ is the optical path of ${I}_{3}$ from AOM3 to the detector. ${f}_{1}$, ${f}_{2}$, and ${f}_{3}$ are the frequencies of beams ${I}_{1}$, ${I}_{2}$, and ${I}_{3}$ respectively.

The phase change of the beam could be detected by measuring the frequency change of the beam. Therefore, the phase change with time can be equivalently regarded as the frequency change of the beam. Detectors 3 and 4 feed their respective signals into the differential frequency calculation module. The differential frequency calculation module calculates the difference between the frequencies of beam ${I}_{1}$ and ${I}_{2}$. The electronic subdivision number of the differential frequency calculation board is $M$. According to the principle of heterodyne interferometry and Eq. (16), the fringe phase change count ${N}_{\varphi }$ can be obtained:

17 $${N}_{\varphi }=M{\int }_{\!\!0}^{t}\left[\left(\frac{{L}_{2}}{{\lambda }_{2}}-\frac{{L}_{1}}{{\lambda }_{1}}\right)+({f}_{1}-{f}_{2})t\right]dt$$

The position of the interference fringe ${L}_{s}$ is expressed as:

18 $${L}_{s}=p\left(\frac{{L}_{2}}{{\lambda }_{2}}-\frac{{L}_{1}}{{\lambda }_{1}}\right)+p\int ({f}_{1}-{f}_{2})dt=\frac{\lambda {N}_{\varphi }}{2M\,\sin \theta }$$

The phase difference control diagram is shown in Fig. 6c. The value output by the system includes the optical path difference caused by the asymmetric installation and adjustment of the exposure system, the optical path difference caused by equipment vibration and air disturbance, and the optical path difference caused by the phase modulation at the previous moment. Equation (18) shows that heterodyne interferometry is a relative measurement method that can be used to measure the change in the phase of the fringe over time. The scanning interference field exposure system splices the exposure based on the superposition of the light spot parts to obtain large aperture holographic grating, so the splicing phase could be controlled by measuring the relative phase of the adjacent fringe.

A phase control experiment is performed by measuring the phase according to the existing optical path of the fringe position measurement. The results of the phase control feedback are shown in Fig. 6d. After static locking, the average fringe phase is 9.713 × 10^−5 ^rad, and the 3*σ* value is 0.349 rad when the grating period is 1740 gr/mm. The displacement change of the interference fringe on the substrate surface is 10.835 nm (3*σ*). After dynamic locking, the average fringe phase is 9.713 × 10^−5 ^rad, and the 3*σ* value is 0.355 rad when the grating period is 1740 gr/mm. The displacement change of the interference fringe on the substrate surface is 11.633 nm (3*σ*).

In summary, a 1500 mm × 420 mm aperture grating with wavefront aberration 0.327*λ* @ 632.8 nm is fabricated by independently proposing a complete set of the scanning interference field exposure system. This system mainly focuses on the high precision measurement of stage displacement, control of interference fringe and real time compensation of grating phase error. It solves the problem that the traditional grating production process cannot be large and fine at the same time. As a core component, the fabricated meter-level size and nano-level precision holographic grating would have potential to be applied in chirped pulse amplification system, high-energy laser and ultra-high precision displacement measurement and other fields.

## Supplementary information

Supplementary information for Controlling the wavefront aberration of a large-aperture and high-precision holographic diffraction grating

## Data Availability

The data supporting this study’s findings are available from the corresponding author upon reasonable request.
