# Supplementary material for: Controlling the wavefront aberration of a large-aperture and high-precision holographic diffraction grating
Source: Light Sci Appl. 2025 Mar 5;14:112. doi: 10.1038/s41377-025-01785-2 (PMC11882855; doi:10.1038/s41377-025-01785-2)
Supplement: Supplementary file 1 — Supplementary information for Controlling the wavefront aberration of a large-aperture and high-precision holographic diffraction grating [file 41377_2025_1785_MOESM1_ESM.docx]

Supplementary Information

**Controlling the wavefront aberration of a large-aperture and high-precision holographic diffraction grating**

**Wenhao Li^1,*^, Xinyu Wang^1,2^, Bayanheshig****^1,*^, Zhaowu Liu^1^, Wei Wang^1^, Shan Jiang^1^, Yubo Li^1^, Shuo Li^1^, Wei Zhang^1^, Yanxiu Jiang^1^, Zheng Wu^1^，Wenyuan Zhou^1,2^**

^1^*Changchun Institute of Optics, Fine Mechanics and Physics, Chinese Academy of Sciences, 130033 Changchun, Jilin, China*

^2^ *University of Chinese Academy of Sciences, 100049 Beijing, China*

* Corresponding Authors

Email: Wenhao Li (liwh@ciomp.ac.cn) and Bayanheshig (bayin888@sina.com)

**Keywords:** large-aperture and high-precision grating, scanning interferometric field exposure system, groove position error, measurement and control

**Supplementary Section 1.** **Control technology for automatic beam alignment**

In the scanning interference system, a millimeter-sized beam is used for exposure, and the degree of beam overlap directly affects the contrast of the interference fringes. It is difficult to meet the required precision in manual adjustment. Therefore, this study combined with the characteristics of the exposure optical path, design a set of beam automatic alignment system. The principle and diagram of the automatic beam alignment system are shown in Fig. s1a and b. The beam position and angle information are obtained through the combined measurement of the decoupling lens and position-sensitive detector (PSD). Here, the conjugate plane of the PSD with respect to the position decoupling lens is defined as the decoupling plane. The PSD is used to detect the position fluctuation of the beam on the decoupling plane, and simultaneously, the angle-sensitive detector (ASD) is used to measure the angle change of the beam on the decoupling plane. The fluctuations information of beam position and angle on this plane and the PSD measurement information are conjugate to each other, so the beam position and angle can be monitored. In the system design and construction, the position of the decoupling plane is made to coincide with the interference field plane to ensure precision in measuring and controlling the interference field. After multi-step adjustment, the beam angle alignment precision is better than 9 μrad, and the beam position alignment precision is better than 10 μm, as shown in Table 1.

In this paper, we calculate the iterative convergence conditions of the beam in the coupling case, and analyze the influences of the alignment and adjustment errors between the mirrors and the decoupling plane in the optical path on the convergence of the system. This is experimentally verified for the alignment path with alignment and adjustment errors. The results show that the designed alignment system effectively adjusts the beam angle and position, and the system has good convergence. The control principle is shown in Fig. s1c. The hardware input/output functions of the PSD, the ASD, and the micro-motion motor are encapsulated into a hardware system server, and data are exchanged through a predefined shared-variable library. The automatic alignment program in the main control computer obtains the information of the PSD and ASD through the USB interface. After being processed by the control algorithm, the micro-motion motor is controlled through the network interface to adjust the optical path. The automatic alignment process is shown in Fig. s1d.

Simulated the influence of alignment error on exposure contrast during a single scan. We assumed a coherent Gaussian beam with a waist radius *ω* of 1 mm, and the alignment errors considered were 10, 100, and 500 μm. The exposure contrasts during a single scan are shown in Fig. s1e where the abscissa represents the radial distribution of the interference field, and the ordinate represents the contrast of the single-scan exposure. When the alignment error is small, the single-scan exposure is close to the ideal value of 1. When the alignment error increases, the contrast of the single-scan exposure decreases, especially at the edge. In Fig. s1f, the abscissa represents the radial distribution of the interference field, and the ordinate represents the exposure of the single scan in the unit of normalized relative intensity. Because of the alignment error, it is obvious that the light intensity of the dark interference fringes is not 0, but there is a certain background light (yellow dotted line), resulting in a decrease in the contrast.

According to the actual exposure requirements, the spacing *s* between two adjacent scans is set to 0.9 mm, the number of steps N is 20 steps, and the grating period is 1800 gr/mm (*p*= 555.6 nm). These parameters were used to simulate the spatial distribution of the exposure contrast for an alignment error *δ* of 10, 100, and 500 μm, as shown in Fig. s1g. The figure shows that when the alignment error is small, such as *δ* = 10 μm, the exposure contrast on the grating substrate surface is relatively uniform and close to the ideal contrast. When the alignment error is large, such as *δ* = 500 μm, the exposure contrast on the grating substrate surface decreases significantly, and simultaneously, the uniformity of the overall exposure on the photoresist surface decreases. With the step-by-step movement of the interference field, ripples appear in the contrast everywhere, indicating that the decrease in contrast is magnified over the entire grating substrate surface through step-by-step scanning.

Table 2. Results for beam position and angle alignment.

| Argument | Maximum value | Standard deviation |
| --- | --- | --- |
| *x* beam position *P_x_* | 13.73 μm | 6.53 μm |
| *y* beam position *P_x_* | 12.97 μm | 6.95 μm |
| Overall directional beam position *P* | 17.03 μm | 9.53 μm |
| *x* beam angle*θ_x_* | 10.13 μrad | 6.54 μrad |
| *y* beam angle*θ_y_* | 12.18 μrad | 5.90 μrad |
| Overall direction beam angle*θ* | 16.17 μrad | 8.81 μrad |


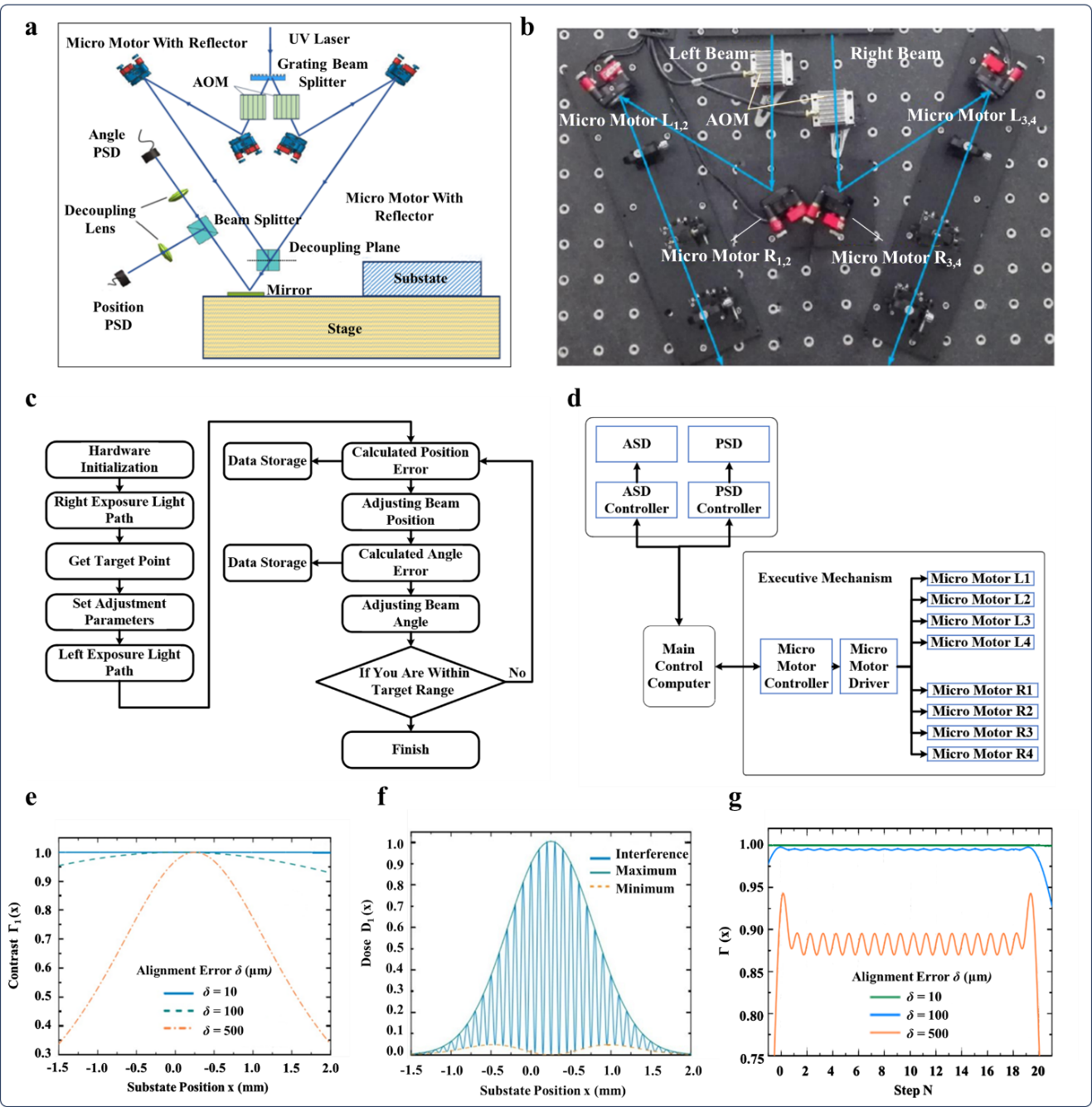


**Fig. s1 Principle of automatic beam alignment**. **a** Diagram of automatic beam alignment. **b** Photograph of the automatic beam alignment system with a superimposed flow diagram. **c** Schematic of the beam alignment controller. **d** Flow chart of the automatic beam alignment system. **e** Effect of alignment error on single-scan exposure contrast. **f** Single-scan exposure. **g** Spatial distribution of exposure contrast for different beam alignment errors.

**Supplementary Section 2. Control technology for beam drift**

Because this technique uses step-by-step scanning and has a long exposure time. The laser beam will drift over time. The beam drift error leads to changes in exposure parameters during the scanning, affects the uniformity of the fabricated mask, and results in poor diffracted wavefront quality. To reasonably suppress the influence of the beam drift error, a beam stabilization system is designed according to the characteristics of this scanning interference system to suppress the drift error. The principle is shown in Fig. s2a. The system mainly includes a laser, a piezoelectric fast-steering mirror (FSM), mirrors, a beam splitter prism (BS), a decoupling lens, and a two-dimensional PSD. After passing through the polarizing beam splitter prism, one laser beam enters the exposure path, and the other beam enters the beam stabilization system. The beam entering the stabilization system passes through two piezoelectric FSMs successively to adjust the beam angle and position postures, and then passes through the beam splitter to divide the beam into two paths for measuring the beam angle and position. The designed measurement system stabilizes the beam as shown in Fig. s2b. The system effectively suppresses the low-frequency drift of the beam. The beam angle precision is better than 1.4 μrad in the *x* direction and is 2.3 μrad in the *y* direction. The total angle precision is better than 2.7 μrad. The beam position precision is better than 3.5 μm in the *x* direction and better than 1.6 μm in the *y* direction. The total position precision is better than 3.9 μm (all are *σ* values), as shown in Fig. s2c.

This paper also obtains the influence of beam angle drift on exposure contrast through numerical analysis, as shown in Fig 4d. The period of the interference field changes linearly with the beam angle drift. The larger the beam angle drift magnitude is, the more drastic the fringe period change is. Under different grating periods, the influence degree of beam drift on the fringe period is also different. The smaller the grating groove density is, the more obvious the influence of beam angle drift on the fringe period is, that is, the smaller the grating period to be fabricated is, the stricter the requirements for beam angle drift are. The influence of different light spot sizes on exposure contrast is shown in Fig 4e. The exposure contrast decreases as the beam angle drift *δ_y_* increases. The larger the beam angle drift magnitude is, the more drastic the decrease in exposure contrast is. Under different exposure light spot sizes, the influence degree of beam drift on exposure contrast is also different. The larger the spot size is, the more obvious the influence of beam angle drift on exposure contrast is. The influence of different light spot drift rates on exposure contrast is shown in Fig 4f. When there is no drift of the beam, the exposure contrast on the photoresist is the ideal exposure contrast of 1. When low frequency drift occurs in the exposure optical path, the exposure contrast on the photoresist decreases as the exposure process proceeds. Under the same number of stepping times, the larger the light spot drift rate is, the larger the decrease range of exposure contrast is.


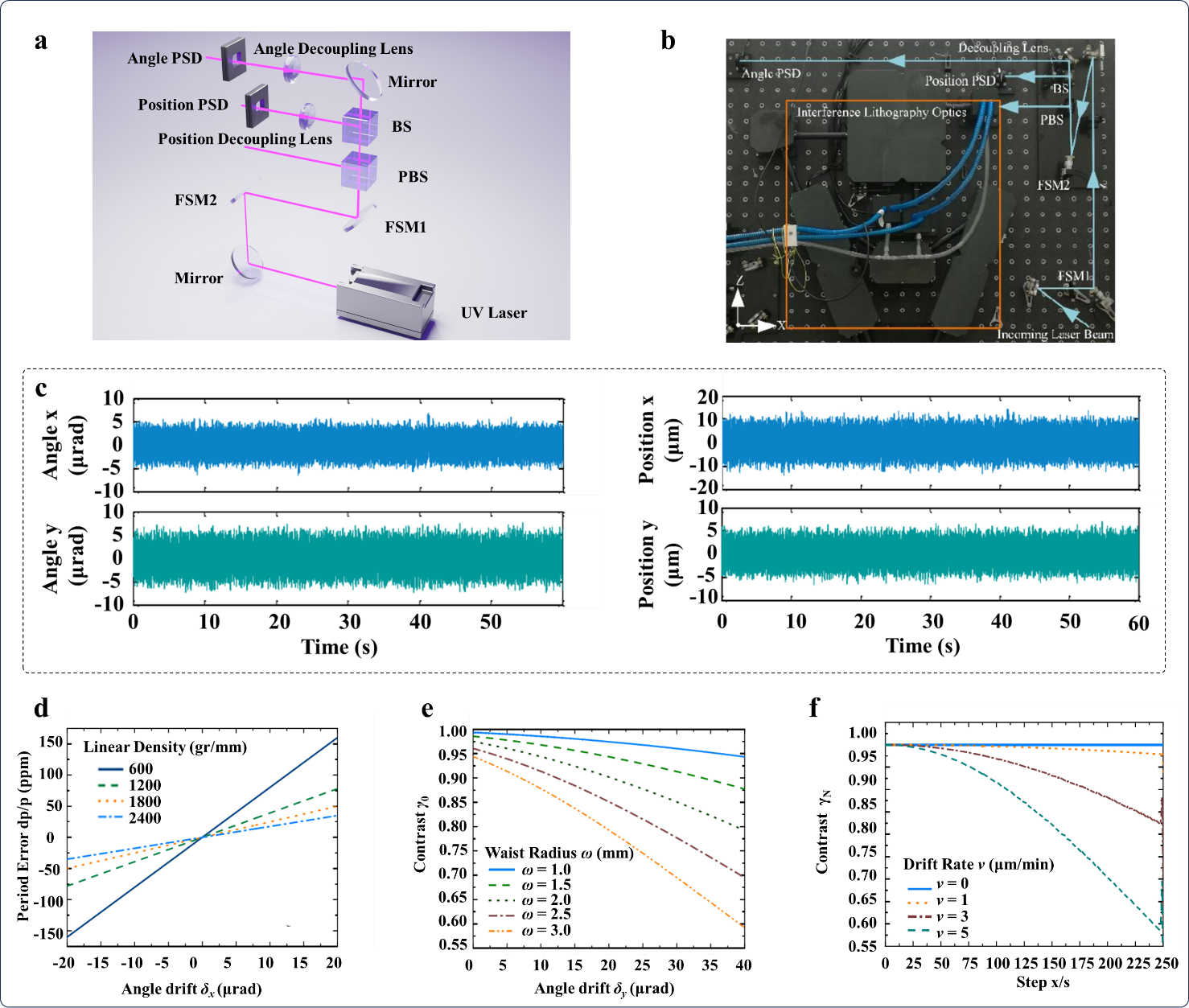


**Fig. s2** **Influence of beam drift control**. **a** Diagram of angular drift. **b** Photograph of the beam stabilization system with a superimposed flow diagram. **c** Beam stabilization results. **d**Influence of angle drift on period error. **b** Influence of angle drift on exposure contrast γ. **e** Influence of spot size on exposure contrast γ. **f** The exposure amount contrast on the photoresist under different light spot drift rates.

## Supplementary Section 3. Model for controlling the grating groove position error (derivation process)

After the laser light is split, the two beams propagate through symmetrical paths with the same beam size and interfere at the beam waist. Therefore, each beam can be regarded as plane light with a Gaussian profile. A coordinate system is established with the origin on the surface of the grating substrate. The electric field distributions of the beams on both sides of *x* can be written in the following forms:

*A*_1_ and *A*_2_ are the respective energy constants of the two beams. *L*_1_ and *L*_2_ are the respective optical paths of the left and right beams from the beam splitting grating to the exposure area. *f*_1_ and *f*_2_ are the frequencies of the left and right beams, respectively. *w* is the spot radius of the two beams at the position of the interference field, and *φ*_0_ is the beam phase constant past the beam splitting grating. The wave numbers are *k*_1_ = 2π/*λ*_1_ and *k*_2_ = 2π/*λ*_2_, and *λ*_1_ and *λ*_2_ are the respective wavelengths of the two beams. The intensity distribution of the coherent superposition of the two light beams is :

where

Suppose the scanning speed in the *y* direction parallel to the interference fringes is *v*, and the scanning path of the *n*^th^ scan is *x*=*S_n_*(*y*). The exposure amount on the photoresist surface on the grating substrate is the sum of the exposures of *N* scans, *D*(*x*,*y*), which is expressed as

$$D\left(x,y\right)=\sum_{n=1}^{N} \int_{-\infty}^{+\infty} I\left(\left[x-S_{n}\left(y\right)\right],y\right)dt=D_{B}\left(x,y\right)\left[1+\frac{D_{A}\left(x,y\right)}{D_{B}\left(x,y\right)}\cos\left(\Phi_{e}\left(x,y\right)\right)\right]$$

where

*D_A_*(*x*,*y*) is the root mean square of the photoresist exposure, *D_B_*(*x*,*y*) is the average exposure on the photoresist, and Φ*_e_* is the exposure phase distribution after *N* scans. After *N* scans, Φ*_e_* is determined by the ruling error distribution of the *n*^th^ scan and the weighting proportion of the adjacent ruling error distribution, and Φ*_e_* can be approximated as the exposure phase distribution of the *n*^th^ scan:

where $p=\lambda/2\sin\theta$ is the grating period, $\left(L_{2}/\lambda_{2}-L_{1}/\lambda_{1}\right)$ is determined by the optical path difference of the two beams, is the phase term related to the frequency modulation of the beams and time, which is what the phase control system adjusts, is the phase change due to nonlinear error. Further, and could be expressed as:

According to in the Eq. (5), the position of the grating groove could be adjusted by controlling the frequency difference between the two beams, so as to compensate the substrate position error and the optical path difference between the beams. Acousto-optic modulator (AOM) is therefore inserted into the optical paths on both sides to adjust the beam frequency. When the modulation frequencies of the two AOMs (AOM1 and AOM2 in Fig. s2) are set to$f_{1v}$ and $f_{2v}$ respectively, the frequencies of left and right beams correspond to $f_{1}=f_{0}+f_{1v}$ and $f_{2}=f_{0}+f_{2v}$ respectively. Therefore, the frequency difference between the two coherent beams is $f_{1v}-f_{2v}$, which could be controlled by AOMs to adjust the position of the grating grooves. If there is an angle *α* between the direction of the interference fringe and the direction of scanning motion is *α*, would be expressed as:

According to Eq. (7), the production of high-precision grating requires high-precision displacement measurement of stage and high-precision control of interference field.

## Supplementary Section 4. -1^st^-order diffracted wavefront of the grating with seamless.

We provide the wavefront data measured by ZYGO interferometer, as shown in Fig.s3. It can be seen that the grating fabricated by us is seamless.


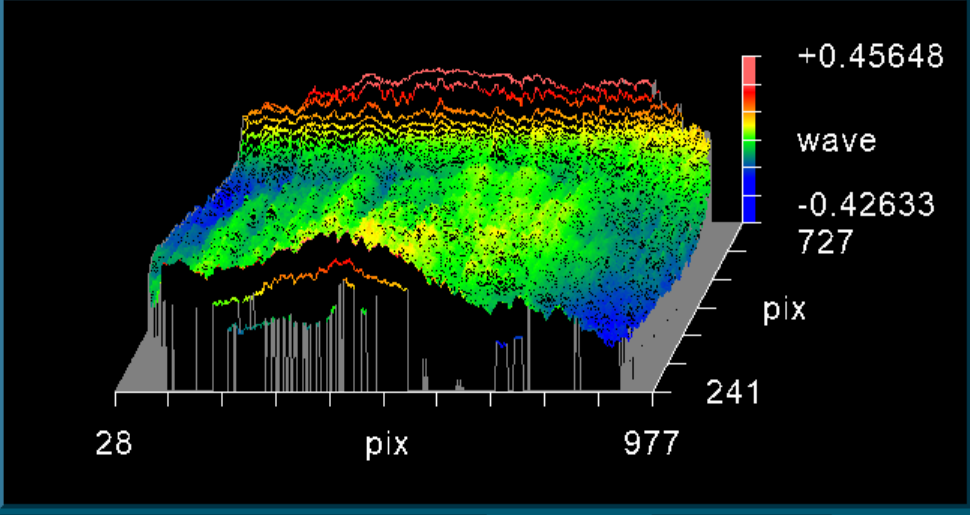


Fig.s3 -1^st^-order diffracted wavefront of the grating.

## Supplementary Section 5. Posture error of integrated displacement measurement system combining grating sensing and laser interferometry

The rotation between the reading head system and the displacement stage has an important effect on the measurement accuracy. Analyzing the influence of the stage angle on the displacement measurement will help to improve the measurement accuracy of the system. Since the stage yaw angle (*θ_x_*) does not affect the measurement accuracy, the influence of the stage pitch angle and roll angle on the measurement accuracy is analyzed in this section.

Pitch angle (*θ_y_*)

The pitch angle of the stage affects the measuring accuracy of the grating sensing but has no effect on the laser interferometer. As shown in Fig. s4, the grating reading head and the exposure point on the grating base are located at the same height. The offset between the grating reference line and the measurement axis is represented by *h*, and the pitch angle error of the stage X axis during movement is represented by *θ_y_*(*x*). Then, the Abbe error in the X direction caused by the pitch angle error during operation is expressed as:

$$\delta_{g}=h\times\tan\theta_{y}(x)$$

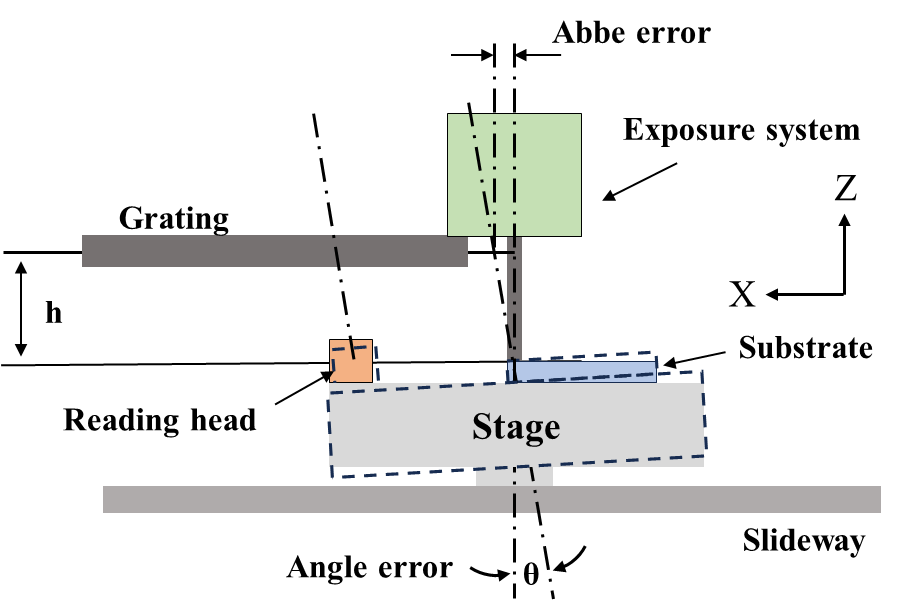


Fig. s4. Stage pitch angle error diagram.

When the system is working, the grating ruler is rigidly connected to the exposure system, and the offset *h* between the grating reference line and the measurement axis is 20 mm. For the stage with a pitch angle of ±1 μrad, the error caused by the offset is ±20 nm.

Roll angle (*θ_z_*)

The stage roll angle affects the measurement accuracy of the laser interferometer but has no effect on the grating sensing. As shown in Fig.5 (a), the stage is in the zero-degree direction, and the X-axis grating sensing measures the distance to the P point on the stage mirror. The coordinate origin of the stage is O. Now, consider rotating the stage around the origin at an angle of *θ_z_*, as shown in Fig.5 (b). If the stage is rotated around the origin (point O), as shown in Fig.5 (b), the point P on the measuring mirror translates *δ_x_* in the X direction and *δ_y_* in the *y* direction. The measuring system now measures the *x*-coordinate of point S.


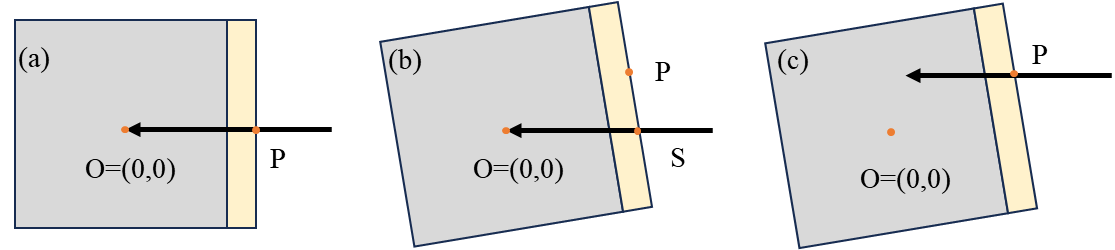


Fig.5 (a) Measuring point P when the stage is not rotating. (b) Stage rotates α at origin O. (c) Stage rotates α around point P.

Considering a property of rotation, a rotation around the origin can be thought of as a rotation around an arbitrary point plus a translation. As shown in Fig.5 (c), the stage rotates around point P. In this case, P is constant for the rotation. The orientation of the stage in Fig.5 (b) can be obtained by performing the rotation in Fig.5 (c) and then translating by (*δ_x_*, *δ_y_*).

We propose a method to determine the position of point P by first rotating the stage around P and then translating the stage by (*δ_x_*, *δ_y_*). The translation by (*δ_x_*, *δ_y_*) is calculated by applying the rotation operator around P and comparing it with the rotation operator applied at the origin O.

Given the rotation angle of the stage *α*, the rotation operator of any vector rotating around the origin of the stage can be expressed as:

$$R_{H}(\alpha)=\left[\begin{matrix} \cos(\theta_{z}) & -\sin(\theta_{z}) & 0 \\ \sin(\theta_{z}) & \cos(\theta_{z}) & 0 \\ 0 & 0 & 1 \end{matrix}\right]$$

Normally, the rotation operator would be a 2 × 2 matrix, but in order to allow for additional translational degrees of freedom, we make a homogeneous transformation of the matrix. The translation operator can be expressed as:

$$T(\delta_{x},\delta_{y})=\left[\begin{matrix} 1 & 0 & \delta_{x} \\ 0 & 1 & \delta_{y} \\ 0 & 0 & 1 \end{matrix}\right]$$

Using two operator forms, the rotation of the point P = (*p_x_*, *p_y_*) can be expressed as *R’*= *T*^-1^(-*p_x_*, -*p_y_*)*R_H_T*(-*p_x_*, -*p_y_*).We assign a frame of reference to point O and a frame of reference to point P. We express the operator operating in the P frame of reference as its equivalent in the O frame of reference. This method is understood as first translating the point P back to the origin by applying *T* (-*p_x_*, -*p_y_*) and then applying the rotation operator (*R_H_*) around the origin. Finally, the translation operator *T* (*p_x_*, *p_y_*) is applied to convert the point P back.

$$R=T(-p_{x},-p_{y})R_{H}T(-p_{x},-p_{y})=\left[\begin{matrix} \cos(\theta_{z}) & -\sin(\theta_{z}) & -\cos(\theta_{z})p_{x}+\sin(\theta_{z})p_{y}+p_{x} \\ \sin(\theta_{z}) & \cos(\theta_{z}) & -\sin(\theta_{z})p_{x}-\cos(\theta_{z})p_{y}+p_{y} \\ 0 & 0 & 1 \end{matrix}\right]$$

Eq. shows that the rotation around the origin can be expressed in another way, as a rotation around P, translated as (*δ_x_*, *δ_y_*). It can be succinctly written as *R_H_* = *T* (*δ_x_*,*δ_y_*)*R*. Then, *δ_x_* and *δ_y_* can be obtained from Eq. :

$$\left\{\begin{matrix} \delta_{x}=\cos(\theta_{z})p_{x}-\sin(\theta_{z})p_{y}-p_{x} \\ \delta_{y}=\sin(\theta_{z})p_{x}+\cos(\theta_{z})p_{y}-p_{y} \end{matrix}\right.$$

Simplifying via cos(*α*) = 1 - *α*^2^/2 and sin(*α*) = *α* yields

$$\left\{\begin{matrix} \delta_{x}=-\frac{\theta_{z}^{2}}{2}p_{x}-\alpha p_{y} \\ \delta_{y}=-\frac{\theta_{z}^{2}}{2}p_{y}+\alpha p_{x} \end{matrix}\right.$$

When the roll angle of the stage is negligible (1 μrad), the second order term can be omitted. Then, the translated *δ_x_* is derived as *δ_x_* ≈ *αp_y_*. If the axis of the X-axis interferometer is aligned with the origin, *p_y_* = 0. If there is an offset of a quantity, *p_y_ = Y_∆_*, the measurement error generated by the stage roll is:

$$Abbe=Y_{\Delta}\theta_{z}$$

In the experiment, for the stage with a roll angle of 1 μrad, we can control the offset within 2 mm by design, and the error caused by the offset is within 2 nm.

Through the calculation of the above errors, the total error of the measurement system can be obtained, as shown in Table s1.

**Table s1. Stage measurement accuracy**

| Error | GI | LI |
| --- | --- | --- |
| Yaw angle (*θ_x_*) | 0 | 0 |
| Pitch angle (*θ_y_*) | ±20 nm | 0 |
| Roll angle (*θ_z_*) | 0 | ±2 nm |
